# Comparison of Treatment Outcomes of Different Immobilized Finger Positions After Repair of Flexor Tendon Rupture in Zones I and II: A Nonrandomized Controlled Trial With Historical Control Group

**DOI:** 10.7759/cureus.62218

**Published:** 2024-06-12

**Authors:** Takaya Tajima, Shiro Yoshida, Hiroki Takashima, Taishiro Kamasaki, Kotaro Jinbo, Koji Hiraoka

**Affiliations:** 1 Hand Therapy, St. Mary’s Hospital, Kurume, JPN; 2 Orthopaedic Surgery, Kurume University School of Medicine, Kurume, JPN; 3 Rehabilitation, Nishikyushu University, Saga, JPN; 4 Orthopaedic Surgery, St. Mary’s Hospital, Kurume, JPN

**Keywords:** postoperative outcome, fixation methods, flexor zone ii, early active mobilization, flexor tendon injury

## Abstract

Introduction

The position of finger immobilization after flexor tendon rupture repair is changed to the extended position to prevent flexion contracture of the interphalangeal (IP) joint. However, in Strickland's assessment, We believe that a reduction in TAF (total active flexion) affects the outcome and that extension fixation is not necessarily the primary focus. For example, there are management methods that swap the fixed position between day and night. It is assumed that some effect is sought by placing the fingers in the flexed position. That is, the method of fixation is currently selected at individual facilities through twists and turns; however, the indications and criteria for selecting finger fixation positions are ambiguous, and they are apparently subject to the experience of therapists. This study aimed to characterize follow-up outcomes of flexion and extension fixation after zones I and II flexor tendon rupture repair.

Methods

This nonrandomized controlled trial with historical controls included 25 patients with flexor tendon ruptures of 30 fingers. The flexion fixation group consisted of 12 patients (n=16 fingers) and the extension fixation group consisted of 13 patients (n=14 fingers). The group with flexion fixation comprised patients who slept with their injured fingers in the flexed position (intervention group). The group with extension was retrospectively selected between April 2017 and March 2019, who slept with their injured finger in the extended position (historical control group). Strickland assessments of the range of motion (ROM) of each joint at the conclusion of hand therapy, the ratio of total active motion of the repaired, to the healthy finger (%TAF), and IP joint extension limitation angles were compared using Mann-Whitney U tests. Ratios of excellent and good ratings based on the Strickland assessment were compared using Fisher exact tests.

Result

The results of the Strickland assessment showed excellent or good outcomes for 22 (73%) of 30 fingers, which was in line with our previous findings. Strickland ratings of excellent were achieved in seven (44%) of 16 fingers and four (28%) of 14 fingers in the groups with flexion and extension fixation, respectively. The outcomes for two (22%) of 16 fingers and seven (78%) of 14 fingers in the groups with flexion and extension fixation were, respectively, rated as good. The proportion of patients rated as excellent was significantly higher in the group with flexion than extension fixation (p=0.040). The %TAF and the active flexion angle of the distal interphalangeal (DIP) joint were higher in the group with flexion than extension fixation (p=0008 and p=0.025, respectively). Furthermore, the total angle of the IP joint limit of extension did not significantly differ between the groups.

Conclusion

Flexion fixation after flexor tendon rupture achieved an excellent Strickland rating and was more effective than extension fixation, especially in terms of the active flexion ROM of the DIP joint. Flexion fixation might be an alternative to extension fixation because the range of flexion should be greater and might provide a range of finger extension motion equivalent to that of extension fixation.

## Introduction

The Kleinert and modified Kleinert methods in which injured fingers are managed in the flexed position have been the most popular immobilization positions after a flexor tendon rupture is repaired [1,2]. Later, extension fixation was used to immobilize injured fingers while asleep and the proximal interphalangeal (PIP) joint at rest [3,4]. These and the development of stronger sutures and exercise therapy have led to fixation in an extended position to prevent flexion contracture and limited extension [5] of the interphalangeal (IP) joint [5,6]. However, decreased total active flexion (TAF) has affected treatment outcomes according to Strickland assessments [7,8]. Extension fixation is not always common, and results after flexion fixation with extension exercises such as changing the position of the fixed finger during the day and night have been good [9,10]. That is, fixation methods are selected by each facility, and we have immobilized fingers in the flexed position after surgical repair of hand flexor tendon rupture since 2019. However, the indications and criteria for selecting the fixation position are unclear and apparently are left to the experience and discretion of therapists. As far as we could ascertain, the outcomes of different immobilization positions at a single institution or under similar post-treatment conditions have never been compared. Furthermore, few studies have compared the outcomes of different fixation positions (flexion and extension) during postoperative care and rehabilitation. This study aimed to determine the final follow-up outcomes of flexion and extension fixation positions after the repair of zones I and II flexor tendon ruptures.

## Materials and methods

Patients

This nonrandomized controlled trial with a historical control analyzed the outcomes of 25 patients with flexor tendon ruptures of 30 fingers that were repaired at our institution between 2017 and 2021.

The inclusion criteria comprised complete deep finger flexor (flexor digitorum profundus, FDP) tendon rupture with or without zones I or II shallow finger flexor (flexor digitorum superficialis, FDS) tendon rupture and started joint range of motion (ROM) training of the operated finger before postoperative day (POD) 7. The exclusion criteria comprised a previous tendon graft or transplant, extensor tendon injuries, fractures, or joint injuries, requiring skin reconstruction, attending other hospitals as outpatients, or being unable to present at our hospital as outpatients at least three times per week for eight weeks postoperatively. Effective April 2019, the hospital changed the fixation position after hand flexor tendon rupture surgery during sleep from the traditional extension position to the flexion position. The group with flexion fixation comprised 12 patients (mean age, 38±18 years) with 16 injured fingers in zones I (n=5) and II (n=11) who slept with their injured fingers in the flexed position (intervention group). The group with extension was retrospectively selected between April 2017 and March 2019. It consisted of 13 patients (mean age, 40±17 years) with 14 injured fingers in zones I (n=4) and II (n=10) who slept with their injured fingers in the extended position (historical control group). This study was registered with the WHO International Clinical Trials Registry Platform (jRCT1070230102, 25/05/2024). Our St. Mary's Hospital Institutional Review Board approved the study (Ken 18-0302), which proceeded according to the ethical principles enshrined in the Declaration of Helsinki (2013 amendment). Consent was obtained via an opt-out period during which patients could decline to participate in the study. However, all approached patients volunteered to participate.

Surgical procedures

All surgeries proceeded under general anesthesia; certified hand surgeons and hand-trained surgeons operated on 19 and 11 fingers, respectively. The FDP was repaired using a FiberWire 4-0 FiberLoop® suture (Arthrex, Naples, FL, USA) in nine fingers using the Yoshizu #1 technique with a six-strand core suture, 15 using the Lim and Tsai method with a six-strand core suture, and six using the Tsuge method with a four-strand core suture [8,11,12]. All FDS were repaired using the Tsuge method using a FiberWire 4-0 FiberLoop® suture (Arthrex) [12]. The epitendinous tendon repair was sutured using the Silfverskiöld method and a simple 5-0 monofilament nylon running stitch [13].

Postoperative therapy and rehabilitation

**Figure 1:**
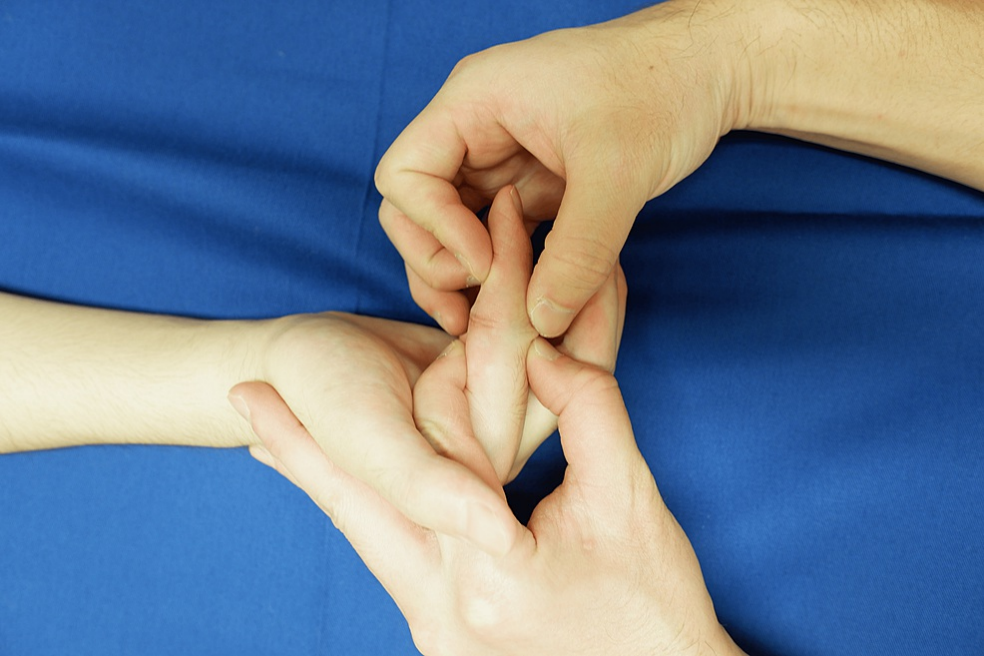
Passive extension exercises according to the Duran method. Patient with zone II finger flexor tendon rupture (index finger). Photographs are shown with consent.

**Figure 2:**
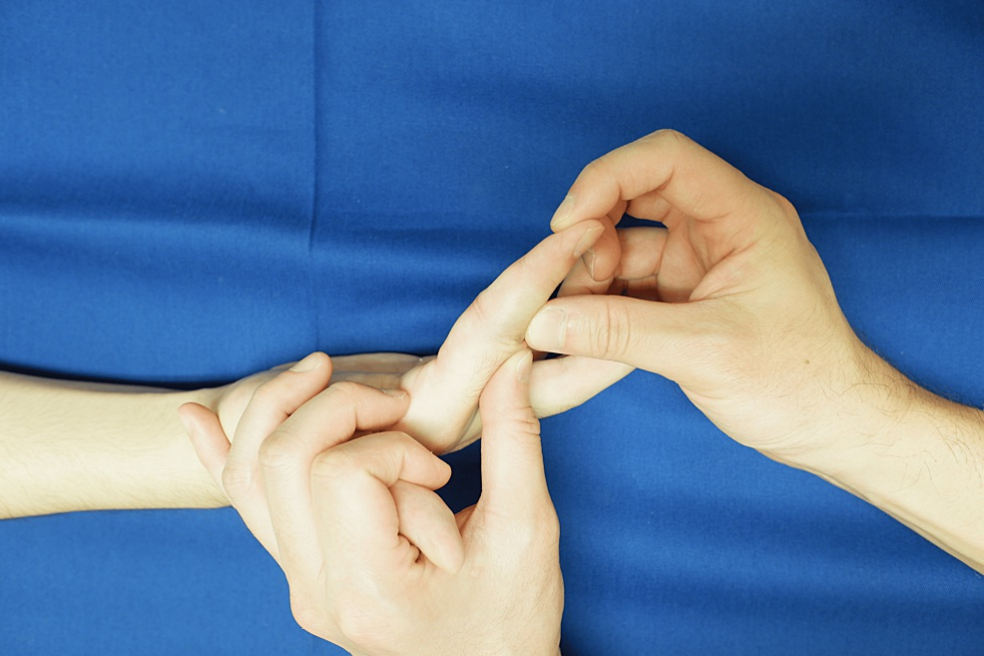
Full extension exercises of the IP joint with the wrist and MP joint in mild flexion. Slight flexion of the MP and wrist joints, and active and passive extension exercises of the PIP and DIP joints. Patient with zone II finger flexor tendon rupture (index finger). Photographs are shown with consent. MP, metacarpophalangeal; IP, interphalangeal; DIP, distal interphalangeal; PIP, proximal interphalangeal

**Figure 3:**
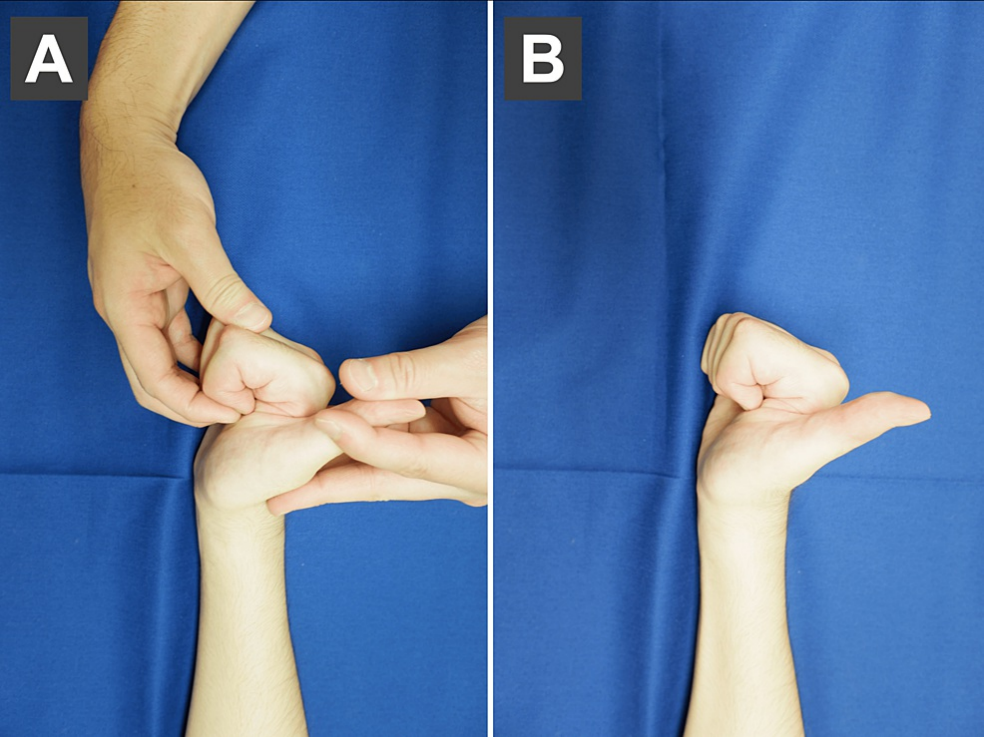
Place and hold in the finger flexion position. (A) Passive flexion exercise of the fingers. (B) Hold in a passively flexed position. Patient with zone II finger flexor tendon rupture (index finger). Photographs are shown with consent.

Three-hand therapists provided postoperative therapy that included starting ROM training for the injured finger joint starting on POD 6. Throughout POD 1-21, the patients executed passive extensions according to the Duran method (Figure 1), with full extensions of the IP joint with the wrist and metacarpophalangeal (MP) joint in mild flexion (Figure 2), and place-and-hold exercises with the finger in the flexion position (Figures 3A, 3B) [14-16].

An extension-restricted resting splint with the wrist at 0° and the MP joint at 30°-60° of flexion was devised by a hand therapist, and fixation within the splint was divided into flexion and extension positions. The patients wore a brace throughout POD 1-21 except for rehabilitation. On POD 21, the patients were allowed to self-administer active flexion and extension movements within the splint. However, splint immobilization was mandatory except for rehabilitation and self-training. Sleep immobilization was continued until POD 56. 

Flexion fixation 

**Figure 4:**
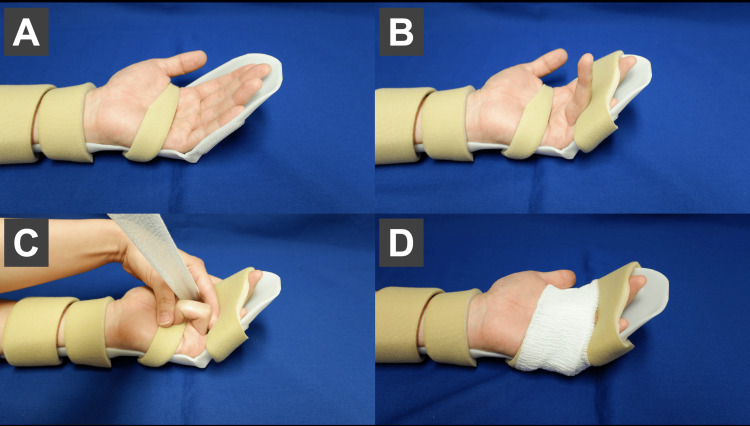
Flexion fixation. Showing the sequence of fixation in the flexed position. (A) The hand therapist will devise an extension-limited resting splint with the wrist at 0° and the MP joint flexed 30° to 60° and fixation within the splint. (B) The operated finger was flexed throughout the day and the adjacent finger was extended with a rest splint (quadriga effect). This inhibits unintended contraction of the FDP and prevents re-tears. (C)The flexion angle of the operated finger was fixed at 70° at the PIP joint and at least 30° at the DIP joint. (D) Fix the finger in a flexed position with a bandage. A healthy subject was photographed as a model, assuming an injury to the left ring finger.

Injured fingers were immobilized using differential splints with the operated finger in flexion throughout the day and the remaining fingers strapped in extension with a resting splint [17]. This uses the quadriga effect and prevents re-rupture caused by unnecessary nighttime clamping [17,18]. The operated finger was fixed in a bent position with a bandage. The flexion angle of the operated finger was fixed at 70° at the PIP joint and at least 30° at the DIP joint (Figures 4A-4D). 

Extension fixation

**Figure 5:**
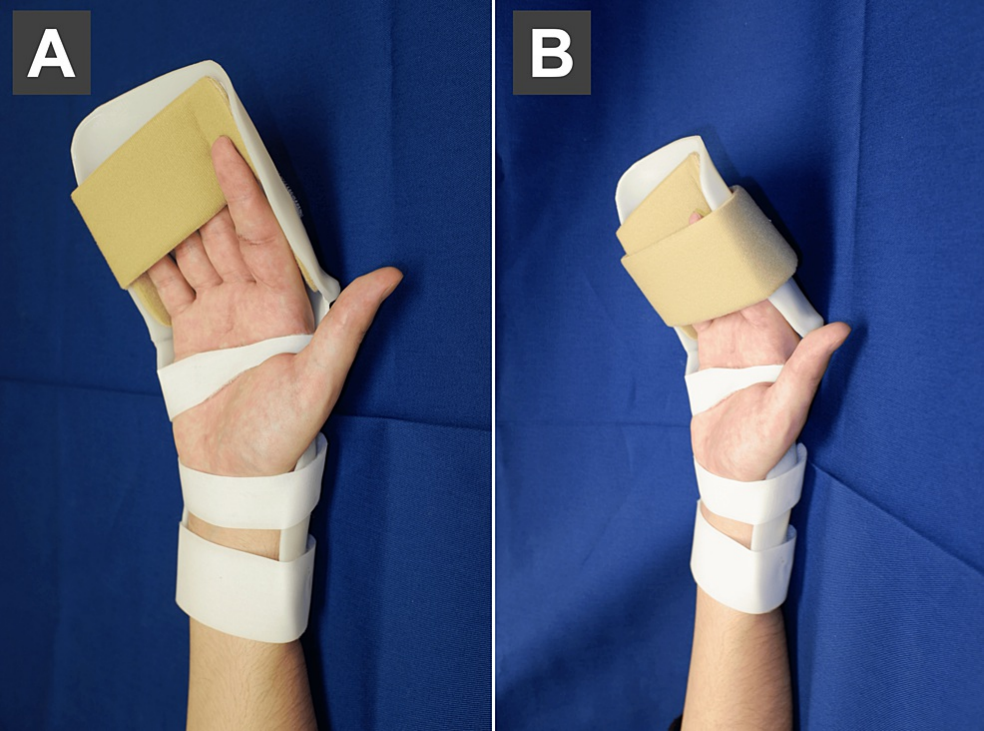
Extension fixation. (A) The wrist joint was fixed at 0°, the MP joint at 30-60° flexion, and the IP joint at 0° extension. (B) Fix with a strap. Patient with zone II finger flexor tendon rupture (index finger). Photographs are shown with consent.

The wrist, MP, and IP joints were fixed at 0°, 30°-60° of flexion, and 0° of extension based on the angle of extension limited by the resting splint (Figures 5A, 5B).

Measurement indices and analytical methods 

Basic and medical information about age, sex (male/female), wait period for surgery (days), injured finger (index/middle/ring/little), number of injured fingers (single/multiple), includes zones I and II, injury type (sharp/blunt), FDS injury (yes/no), volar plate injury (yes/no), artery injury (yes/no), nerve injury (yes/no) were extracted from medical records. The measured indicators were the ROM of each joint, the ratio of TAF in the repaired, to the contralateral healthy finger (%TAF), the total extension limit angle of the IP joint, and the Strickland rating at the endpoint. The ratio of the TAF on the repaired to the contralateral healthy finger was calculated by summing the degrees of active flexion angles of the MP, PIP, and DIP joints on the repaired side. The total extension limit angle of the IP joint was determined by summing the active extension limit angles of the PIP and DIP joints. The Strickland ratings were excellent and good if the TAM of the IP joint was ≥150° and ≥85%, 125°-149°, and 70%-84% compared with the healthy side. The evaluation was also considered acceptable or unacceptable if the TAM and the ratio to healthy contralateral finger were, respectively, 90°-124°, 50%-69%, and <90° and <50%. The patients were assigned to groups based on whether their fingers were immobilized by flexion or extension fixation. We then compared variables thought to influence the course of treatment using χ², Fisher exact, and Wilcoxon tests.

## Results

The analysis included 25 people with 30 injured fingers that were immobilized by flexion or extension fixation. Table 1 shows that none of the variables that might affect treatment outcomes significantly differed between the groups. The Strickland ratings of 22 (73%, excellent and good) of 30 fingers were rated excellent with seven (44%, n=16 fingers) and four (28%, n=14 fingers) fingers in the groups with flexion and extension fixation, respectively. The two (22%, n=16 fingers) and seven (78%, n = 14 fingers) in the flexion and extension fixation groups, respectively, were rated as good (Table 2). Thus, the proportion of patients rated as excellent was significantly higher in the group with flexion than extension fixation (p=0.040; Table 2). The %TAF ratio of the injured to the contralateral healthy finger was significantly higher in the group immobilized with flexion than extension fixation (94%±6% vs. 85%±10%; p=0008). The active flexion angle of the DIP joint was higher in the group with flexion than extension fixation (63°±19° vs. 35°±15°; p=0.025). The total angle limit of IP joint extension did not significantly differ between the groups (Table 3).

**Table 1:** Patient demographics. Median (25% tile-75% tile); Wilcoxon test. *Fisher's exact probability test ^†^χ2 test FDS, flexor digitorum superficialis

Index		Flexion fixation group (n=12 people, 16 fingers)	Extension fixation group (n = 13 people, 14 fingers)	p-value
Age	(year)	33 (22-56)	37 (24-55)	0.689
Sex (male/female)*	(people)	11 (92%)/1 (8%)	12 (92%)/1 (8%)	0.740
Surgical waiting period	(day)	5 (1-7)	4 (2-8)	0.728
Digit*				
Index	(fingers)	6 (37%)	8 (57%)	0.512
Middle	(fingers)	5 (31%)	1 (7%)	
Ring	(fingers)	2 (13%)	1 (7%)	
Little	(fingers)	3 (19%)	4 (29%)	
Number of injured fingers (single/multiple)*	(people)	8 (67%)/4(33%)	12 (92%)/1 (8%)	0.136
Zone injury (I/II)*	(fingers)	5 (31%)/11 (69%)	4 (29%)/10 (71%)	0.596
Type of injury (sharp/blunt)*	(fingers)	11 (69%)/5 (31%)	12 (86%)/2 (14%)	0.256
FDS injury (yes/no)^†^	(fingers)	6 (37%)/10 (63%)	7 (50%)/7 (50%)	0.491
Damage of the volar plate (yes/no)*	(fingers)	11 (69%)/5 (31%)	11 (79%)/3 (21%)	0.426
Arterial injury (yes/no)*	(fingers)	10 (63%)/6 (37%)	12 (86%)/2 (14%)	0.154
Nerve injury (yes/no)^†^	(fingers)	6 (37%)/10 (63%)	10 (71%)/4 (29%)	0.063

**Table 2:** Proportion of excellent and good in the Strickland ratings. n (%); Fisher's exact probability test. Flexion fixation had a higher percentage of excellent by Strickland ratings than extension fixation. *p<0.05

Strickland ratings	Flexion fixation group (n=16)	Extension fixation group (n=14)	p-value
Excellent	7 (44%)	4 (28%)	0.040*
Good	2 (12%)	9 (64%)	

**Table 3:** Comparison of clinical evaluation of the final follow-up. Median (25% tile-75% tile); Mann-Whitney test *p<0.05 ^†^p<0.01 POD, postoperative day; TAF, total active flexion

Index		Flexion fixation group (n=16)	Extension fixation group (n=14)	p-value
%TAF	(%)	94 (88-98)	85 (82-88)	0.008^†^
Sum of extension limiting angles	(°)	-5 (-14--4)	-2 (-15-0)	0.208
DIP joint active flexion	(°)	63 (39-74)	35 (30-50)	0.025*
PIP joint active flexion	(°)	90 (85-101)	100 (98-103)	0.101
DIP joint active extension	(°)	0 (-4-0)	0 (-11-0)	0.377
PIP joint active extension	(°)	-5 (-10-0)	0 (-3-0)	0.052
PODs follow-up	(day)	164 (130-188)	135 (97-187)	0.376
Days spent in hospital	(day)	20 (12-26)	26 (20-31)	0.112

## Discussion

We compared the outcomes after flexor tendon rupture repair of fingers immobilized by fixation at different angles. First, the percentages of "excellent" and "good" as rated by Strickland were compared to previous studies and found to be comparable (Table 4). Of note in this study, significantly more patients with flexion fixation received an "excellent" rating by Strickland; flexion ROM, %TAF, and active flexion angle of the DIP joint were significantly greater with flexion fixation than with extension fixation, but the IP joint extension restriction angle totals were not significantly different between the groups. The reason for greater flexion ROM in the fingers immobilized by flexion fixation was the characteristics of this position. 

**Table 4:** Comparison of treatment outcomes using the Strickland ratings. The percentages of excellent and good by Strickland ratings were compared with previous studies. This study also obtained comparable results.

Author		Strickland ratings				Re-injury
		Excellent	Good	Fair	Poor	
Zhou et al. [19] (n=50)	n (%)	30 (58%)	13 (24%)	3 (5%)	0 (0%)	0 (0%)
Moriya et al. [20] (n=148)		80 (54%)	39 (26%)	17 (11%)	11 (7%)	0 (0%)
Peck et al. [21] (n=76)		6 (4%)	17 (23%)	28 (38%)	24 (33%)	3 (7%)
Giesen et al. [22] (n=32)		66 (18%)	6 (22%)	2 (7%)	1 (14%)	0 (0%)
This study (n=30)		11 (36%)	11 (36%)	8 (27%)	0 (0%)	0 (0%)

In extension fixation, the tendon sutures are adhesion at the distal sliding position. Therefore, proximal sliding of the tendon by active flexion movement is necessary for adhesion release. The tendon gliding distance during active flexion exercises ranges from 11 mm to 30 mm, but postoperative edema and pain are associated with a 15% increase in resistance during flexion exercises and a decrease in gliding distance [23-26]. After fixation in the extended position, resistance occurs during flexion movements, and acquiring a flexible ROM takes time. Adhesion dissociation after extension fixation depends on active flexion movements, as well as the swelling and pain thresholds of patients; thus, the results might not be consistent [26]. In contrast, flexion fixation involves flexion of the PIP and DIP joints of an injured finger by 70° and >30°, respectively [27]. This should cause a proximal slide of the FDP of the PIP joint of 3.25 mm and adhesions so that distal sliding of a repaired tendon is achieved for better dissection [28]. Considering adhesion dissociation after fixation, a cadaver study found that the FDP slid by 8.2 mm during other extension movements of the PIP and DIP joints at 60° MP joint flexion [24]. These findings indicated that adhesion dissociation was sufficient during other extension movements after flexion fixation. We acknowledge that therapists can manage this passive extension exercise load according to the Duran method [14]. After fixation in the flexed position, the injured finger spends less time in passive flexion movements. Active flexion place-and-hold movements are easy to execute and might help to prevent extension contracture and minimize the amount of force required for active flexion [9,15-17]. Therefore, we consider that dissections of adhesions are more stable and flexion movements more efficient after fixation in the flexed than in the extension position. Thus, to interpret ROM differences between the groups, an understanding of the different adhesion positions of tendon repair sites, finger fixations, and tendon sliding methods is required to dissect adhesions [23]. The present findings indicate that the range of flexion motion should be greater after flexion, than extension fixation, and also provide a range of finger extension comparable to that of extension fixation. 

Careful consultation with a physician is essential when introducing flexion fixation, which allows an injured finger to be managed while flexed, thus reducing the tensile load on the repaired tendon [29]. The application of a limb position compatible with the quadriga effect is also advantageous in terms of protecting the repaired tendon, as it can prevent re-rupture due to unintentional gripping [17,23]. On the other hand, great care must be taken to prevent flexion contractures, and ROM training by a therapist is crucial and must be ensured. This might not be suitable for outpatient management. Furthermore, the risk of re-tear is important to manage while doing extension exercises after flexion fixation. We, therefore, recommend that therapists personally confirm the maximum range of extension motion intraoperatively. In addition, estimated maximal loads of 12 N and 9 N are applied to the FDP and the FDS, respectively, when the fingers are fully extended in the middle position of the wrist joint [30]. The FDP and FDS are indicated when using a four-strand or greater core suture.

Previous reports of flexor tendon repair only describe outcomes with either flexion or extension immobilization because postoperative tendon repair protocols are often defined by individual institutions. Therefore, the strength of the present study is that the postoperative treatment and rehabilitation after flexor tendon repair of the hand were unified, and the outcomes of different fixation finger positions could be compared. A limitation of this study is that treatment outcomes were only investigated at the end of the study. Thus, the influence of fixed finger positions on changes in angles over time could not be discussed.

Nonetheless, this study revealed differences in outcomes according to the positions of immobilized fingers and might contribute to the choice of external fixation while asleep.

## Conclusions

This study revealed differences in outcomes after flexor tendon rupture repair depending on the position of the fingers to be immobilized. Flexion fixation after flexor tendon rupture achieved an excellent Strickland rating and was more effective than extension fixation, especially in terms of the active flexion ROM of the DIP joint. Flexion fixation might be an alternative to extension fixation because the range of flexion should be greater and might provide a range of finger extension motion equivalent to that of extension fixation. In the future, the influence of the position of the fixation finger on the transition of ROM needs to be clarified. In addition, knowledge of the characteristics of each fixation position may lead to the selection of an appropriate fixation position according to the progress after flexor tendon rupture repair.
